# Acute normobaric hypoxia as a reversible probe of oxygen-limitation contributions to cognitive aging

**DOI:** 10.1016/j.nbas.2026.100161

**Published:** 2026-07-14

**Authors:** Pierrick Laulan

**Affiliations:** Institute of Psychology, University of Lausanne, Bâtiment Géopolis, 1015, Lausanne, Switzerland

**Keywords:** Acute hypoxia, Cognitive aging, Cerebral oxygenation, Associative memory, Cerebrovascular reserve, Oxygen extraction fraction

## Abstract

Chronological age cannot be experimentally manipulated, which limits causal tests of candidate mechanisms in cognitive aging. This focused review evaluates acute, dose-controlled normobaric hypoxia as a reversible probe of whether oxygen-delivery and cerebrovascular-reserve constraints contribute to age-sensitive cognitive phenotypes, particularly associative episodic memory. Evidence from cognitive hypoxia, cerebrovascular aging, and cerebral oxygenation suggests acute hypoxia and later-life cerebrovascular compromise share common mechanisms that reduce the capacity to meet increased neural metabolic demands during high-load cognitive tasks. Associative memory is proposed as a particularly informative target phenotype because it is reliably vulnerable in aging, strongly dependent on hippocampal binding, and theoretically dissociable from more global performance decrements. Emotional effects are considered only insofar as arousal and sustained stress can modify associative binding, with acute hypoxia framed as imposing conditions of vulnerability under sustained and unavoidable stress rather than reproducing age-related positivity. Four conditions are then identified for informative oxygen-limitation inference: dose and capnic (arterial or end-tidal carbon dioxide) characterization, phenotype-specific dissociation tests, coupling to oxygen-sensitive markers, and discriminant validity against non-specific changes in stress, discomfort, or fatigue. Acute normobaric hypoxia is therefore presented as a reversible causal probe, not as a model of aging; its value depends on stringent boundary conditions and careful mechanistic interpretation.

## Highlights

• Acute hypoxia is framed as a reversible probe of oxygen-limited aging.
• Associative memory is proposed as an especially informative phenotype.
• Cerebral oxygenation and neurovascular reserve form the main bridge.
• Sustained-stress vulnerability clarifies affective boundary conditions.
• Valid inference requires dose, capnia, mechanism, and discriminant tests.

## 1 Introduction

Aging neuroscience faces a recurring challenge: chronological age cannot be experimentally manipulated, which limits causal tests of candidate mechanisms. Observational comparisons, including longitudinal designs, are indispensable for describing age-related and within-person changes, but they remain vulnerable to cohort effects and practice or retest effects and therefore cannot, on their own, disentangle causal pathways from correlated change [1], [2]. This limitation motivates complementary approaches that transiently perturb a candidate physiological constraint, quantify selective cognitive signatures, and test whether those signatures covary with mechanistic readouts within-person. Throughout, “cognitive aging” refers primarily to typical age-related change in cognitively normal adults unless cerebrovascular or neurodegenerative pathology is explicitly discussed.

This article takes the form of a focused, mechanism-oriented review. Its aim is to evaluate whether acute normobaric hypoxia can serve as a reversible probe of oxygen-limitation contributions to cognitive aging, and under what boundary conditions (i.e., the conditions that must hold for the inference to be defensible) it would do so. It is not intended as an exhaustive survey of the hypoxia-cognition literature.

## 2 Oxygen-delivery and cerebrovascular-reserve constraints as a candidate aging pathway

### 2.1 Oxygen availability as a plausible constraint

Neural activity is energetically costly. Despite accounting for a small fraction (approximately 2%) of body mass, the brain consumes a disproportionate share (approximately 20%) of resting oxygen [3], [4], [5]. Across adulthood, cerebrovascular supply and oxygen metabolism show measurable shifts. Large MRI studies concurrently estimating cerebral blood flow (CBF), venous oxygenation, and the cerebral metabolic rate of oxygen (CMRO₂) report age-related reductions in CBF together with lower venous oxygenation, while the direction and magnitude of resting CMRO₂ effects remain more debated across modalities and analytic pipelines [6], [7]. In simplified terms, CMRO₂ is jointly determined by CBF, oxygen extraction fraction (OEF), and arterial oxygen content (CaO₂). These observations can be situated within an established hemodynamic framework in which reductions in cerebral perfusion are first buffered by autoregulatory vasodilation (Stage I) and, when this capacity is exhausted, by increases in OEF (Stage II) that help preserve CMRO₂. Resting OEF is approximately 0.40 [8] and cannot approach unity, because capillary transit heterogeneity and tissue oxygen tension constrain maximal extraction [9]. Estimates from maximally vasodilated tissue place the extraction ceiling near 0.53 [10], so only when this oxygen extraction reserve is exhausted does metabolic compromise ensue [11].

Recent work suggests that this Stage II-like compensation may be especially relevant in older adults with greater cerebrovascular burden (i.e., the cumulative load of small-vessel and microvascular pathology, including arteriolar stiffening, microvascular rarefaction, and white-matter hyperintensities). Using simultaneous PET-MRI, McFadden et al. [12] reported that impaired blood supply and longer arterial transit time were associated with compensatory OEF increases while CMRO₂ remained preserved. In a large community-based cohort, Yan et al. [13] further showed that the high-OEF/low-CBF pattern was associated with the most adverse profile, with greater white matter hyperintensity burden and poorer cognition, whereas higher CBF appeared to buffer vulnerability even when OEF was elevated. Aging should therefore not be treated here as a uniform hemodynamic Stage II state. The central proposal is narrower. Some later-life trajectories, especially those with greater vascular burden, may involve a reduced cerebrovascular reserve that becomes most visible when transient metabolic demand increases. Complementary reserve-sensitive measures such as cerebrovascular reactivity may further sharpen interpretation of vascular headroom (i.e., the margin between resting cerebral oxygen delivery and the maximum the system can sustain) in older adults [14]. Preclinical work is also consistent with this possibility, suggesting that aging can compromise microvascular oxygen delivery and increase the prevalence of hypoxic microdomains in mouse cortex [15], [16].

### 2.2 Acute normobaric hypoxia as a reversible stress test

If oxygen delivery or oxygen utilization constrains selected cognitive phenotypes in aging, experimentally reducing oxygen availability in young adults should produce at least partial overlap with some age-sensitive signatures. Acute normobaric hypoxia is relevant to this question because it transiently lowers inspired oxygen under laboratory control while remaining reversible and titratable. Conceptually, acute normobaric hypoxia is best understood as a stress test of cerebrovascular reserve, the capacity to maintain adequate cerebral oxygen delivery when neural demand increases, and not as a direct manipulation of neuronal oxygen consumption. Because hypoxemia induces compensatory vasodilation and changes in ventilation, any useful inference must remain focused on oxygen-delivery and reserve constraints unless direct metabolic measures are available [17], [18], [19], [20].

Several properties nevertheless make acute normobaric hypoxia attractive as a reversible probe. First, the manipulation is dose-controllable. Researchers can specify the inspired oxygen fraction (FiO₂) and continuously verify the achieved challenge through peripheral oxygen saturation (SpO₂). Second, brief exposure effects are often at least partially reversible after reoxygenation, although recovery kinetics can vary as a function of endpoint and dose [21]. Third, individual differences in hypoxic sensitivity can be quantified instead of treated as unobserved noise. Because the label “acute” is used inconsistently across fields, the term is used here to refer to brief, dose-controlled laboratory exposures lasting from minutes to hours, consistent with the cognitive hypoxia literature [22], [23]. A recent meta-analysis of this literature confirms a moderate overall impairment of cognition under acute hypoxia and, of direct relevance to a normobaric probe, finds the effect somewhat larger under normobaric than under hypobaric conditions [24].

## 3 Associative episodic memory as an informative target phenotype

### 3.1 Associative memory under acute hypoxia and in aging

Associative (relational) memory refers to memory for the links between the elements of an experience, such as an item and its context or a face and a name, not the individual elements in isolation. Forming and retrieving these links relies on hippocampal binding (e.g., [25]). Meta-analytic evidence indicates that acute hypoxia impairs cognitive performance overall, including memory-related outcomes [23]. A more recent simulated-altitude meta-analysis reported a large pooled impairment for memory, although this estimate was based on a limited number of studies and displayed substantial heterogeneity [26]. A larger meta-analysis refines this picture. Under acute hypoxia, response accuracy is impaired more than speed, and the accuracy cost is unevenly distributed across domains, concentrated in attention, executive function, memory, and learned knowledge, with carbon dioxide a significant moderator [24]. Importantly, the hypoxia literature has relied predominantly on short-term or working-memory tasks and on composites probing memory for isolated information, whereas far fewer studies have directly examined episodic associative binding [27], [28], [29]. This gap matters because relational binding depends critically on hippocampal circuitry [25], [30], [31], and emerging evidence, primarily from rodent work, suggests that hippocampal networks can show sensitivity even to mild short-term oxygen perturbations [32].

Associative episodic memory is also a privileged target phenotype from the perspective of aging. Episodic memory decline is a prominent feature of healthy aging, and age effects are reliably larger for associative memory than for item memory [33], [34], [35]. Attention, processing speed, and strategic/executive control also decline with age and contribute to relational encoding and retrieval; therefore, age-related associative deficits should not be assumed to represent pure binding impairments, because broader age-related changes in processing resources may also contribute to performance costs [33], [36], [37], [38]. The associative deficit hypothesis was advanced in part to address this uncertainty. The age deficit is disproportionately larger for associations than for the constituent items, persists once item memory is taken into account, and is only partly reduced by supportive encoding, a pattern that general slowing alone does not readily explain [34], [35]. The effect is therefore most informative when tested as a condition-by-memory-type interaction, not as a single associative score (see Section 5.2). This dissociation gives the present argument its specificity. A useful oxygen probe should not merely produce global cognitive slowing or generic task difficulty; it should preferentially affect a phenotype that is already theoretically and empirically linked to aging. Associative memory satisfies that requirement better than a broad “memory impairment” label because it is hippocampus-dependent, dissociable from more familiarity-based item recognition, and strongly tied to binding operations that are vulnerable in later life [39], [40], [41].

### 3.2 Emotional arousal as a boundary condition for associative memory

Emotional material bears on the present argument that binding-dependent associative memory is the most diagnostic target phenotype for an oxygen-limitation probe. Because emotional arousal elicits its own dissociations, emotions complicate interpretation of item-versus-associative dissociations. Arousal prioritizes the encoding of individually salient items, which strengthens item memory, while leaving the binding of those items to one another or to their context unchanged or even impaired [42], [43], [44], [45], [46]. At the behavioral level this emotional binding deficit resembles the expected profile of an oxygen-limitation mechanism, namely relative sparing of item memory alongside a disproportionate relational binding deficit, yet it arises from attentional prioritization instead of an energetic constraint. Because emotional arousal can reproduce the diagnostic signature through an unrelated mechanism, emotional material is most useful as a boundary-condition test, in that it distinguishes the predicted oxygen-limitation dissociation from an arousal-driven one rather than serving as an independent target.

Two consequences follow. First, acute hypoxia can itself alter affective state, but the direction and magnitude of these mood effects vary substantially with dose, duration, and context [47], [48], [49], so affect is an additional nuisance variable to be taken into account, not a dependable property of the manipulation. Second, acute hypoxia should not be construed as reproducing the age-related positivity effect, i.e., the tendency of older versus younger adults to favor positive over negative information in attention and memory [50], [51]. This effect is commonly understood as a goal-directed regulatory bias that draws on intact cognitive-control resources, and it is accordingly most evident when controlled processing is possible [52], [53]. Because acute hypoxia constrains rather than supports those control resources, it should weaken this regulatory advantage instead of shifting performance toward positivity [54], [55], [56]. The two accounts predict different affective profiles, and a positivity-like pattern under hypoxia would count against an oxygen-limitation interpretation rather than for it.

Taken together, affective endpoints are best treated as interpretive boundary conditions for associative-memory inference, that is, as constraints the oxygen-limitation signature must survive, and not as an autonomous explanatory axis.

## 4 Mechanistic bridge between acute hypoxia and cognitive aging

### 4.1 Cerebral oxygenation and cerebrovascular reserve

During acute hypoxia, cognitive change can co-occur with measurable changes in cerebral hemodynamics and oxygenation, but those signals need not be monotonic because hypoxemia induces compensatory increases in CBF that can partially preserve oxygen delivery despite reduced arterial oxygen content [19], [20]. In a field high-altitude design, Davranche et al. [57] observed early slowing and increased decision errors shortly after arrival at 4350 m, followed by increased prefrontal functional near-infrared spectroscopy (fNIRS) responses over subsequent days, compatible with compensatory microvascular recruitment. In controlled graded normobaric hypoxia, Williams et al. [58] showed that performance change covaried with SpO₂ and cerebral oxygenation, whereas sympathoadrenal and hypothalamic-pituitary-adrenal markers were not predictive of cognitive change. This pattern is important because it suggests that cerebral oxygenation may constitute a more proximal predictor of cognitive vulnerability than peripheral stress markers, even though compensatory dynamics complicate simple dose-response interpretations.

In aging, converging vascular and metabolic evidence points to narrower oxygenation headroom. Lifespan MRI studies report reduced CBF and lower venous oxygenation with age, while the impact of aging on resting CMRO₂ remains debatable [6], [7]. Regionally, older adults also show reduced resting hippocampal perfusion linked to memory [59]. Complementary PET evidence suggests age-related reductions in global brain oxygen consumption and links episodic memory performance to hippocampal and thalamic resting oxidative metabolism [60]. Age-related degradation of neurovascular coupling may further constrain oxygen delivery during cognitive demand [61], [62]. Despite their distinct etiologies (exogenous oxygen restriction in hypoxia vs. endogenous vascular and metabolic decline in aging), both states may therefore reduce the capacity to meet transient neural metabolic demand during high-load cognition, particularly in hippocampal circuits supporting associative binding.

### 4.2 Secondary convergent signatures

Aside from cerebral oxygenation, convergent signatures (i.e., additional physiological correlates that shift in the same direction under both acute hypoxia and aging) may exist, but should be considered secondary in the present discussion. Acute hypoxia can reduce heart-rate variability (HRV), although effects vary across protocols [63], and aging is likewise characterized by robust HRV decline, with lower vagally mediated indices tending to be associated with poorer cognition, especially executive control [64], [65], [66]. Hypoxia may also engage cortisol responses in a dose-dependent manner [67], [68], while age-related cortisol profiles are heterogeneous and should be interpreted cautiously [69], [70], [71]. Similar caution applies to electrophysiological signatures, whose relation to oxygen limitation depends strongly on hypoxia severity and task context [72], [73], [74]. These secondary convergences may help characterize individual vulnerability, but they do not substitute for phenotype-specific cognitive dissociations and oxygen-sensitive mechanistic markers.

Convergences across associative memory, cerebral oxygenation, and these secondary signatures suggest that acute hypoxia and later-life cognitive aging may share sensitivity to oxygen and vascular constraints. However, parallel patterns across levels of analysis do not establish that hypoxia can serve as a valid experimental probe of oxygen-limited aging mechanisms. Without explicit criteria specifying what would count as evidence for, or against, an oxygen-limitation contribution, observed parallels remain correlational and mechanistically ambiguous. The next section therefore identifies four conditions under which inference from acute hypoxia to oxygen-limited components of cognitive aging becomes theoretically informative.

## 5 Conditions for interpreting oxygen-limitation effects

The proposed framework focuses on associative or binding-dependent episodic memory because this phenotype offers the clearest bridge between aging and transient oxygen challenge. The aim is not to prescribe a single experimental design. Rather, the following conditions define the minimum interpretive requirements under which convergence between acute hypoxia and cognitive aging would become theoretically meaningful rather than merely descriptive.

### 5.1 Dose control, capnic context, and reversibility

For the probe to be interpretable, the oxygen challenge must be characterized in terms of the achieved physiological challenge, not only the intended settings. Hypoxic dose, exposure duration, barometric context, and the time course of desaturation and recovery matter because they alter the physiological cascade and can change which endpoints are affected [21], [23], [75], [76]. Reversibility is equally central. If acute hypoxia is used as a reversible probe, cognitive and physiological effects should track exposure and, after reoxygenation, recover on a time course plausible for the studied endpoint. What counts as a plausible time course should be specified in advance from the hypothesized mechanism. An oxygen-delivery effect should resolve as peripheral and cerebral oxygenation normalize, whereas slower or more variable recovery would implicate additional processes. This expectation should be flexible, because recovery after acute hypoxia is itself heterogeneous and not yet predictable, with some cognitive and physiological indices normalizing quickly and others lagging behind reoxygenation [21]. Expected recovery windows are therefore best defined in advance for each endpoint and treated as an empirical question. A second requirement concerns capnic context. Because hypoxia commonly changes ventilation and the end-tidal partial pressure of carbon dioxide (PETCO₂), oxygen-limitation inference weakens unless oxygen-content effects can be distinguished from CO₂-related hemodynamic shifts [77], [78], [79], [80].

To make these parameters concrete, Table 1 summarizes a representative, non-exhaustive selection of acute-hypoxia protocols from the cognitive literature, including the hypoxic dose, exposure duration, the abruptness of hypoxia onset and reoxygenation, the study population, the targeted cognitive domain, key outcomes, and whether capnic status was characterized. Three features are notable for the present purpose. First, almost all such studies have been conducted in young, healthy adults; the oldest cognitive data come from middle-aged adults, in whom executive and working-memory costs emerge at a milder hypoxic dose than in younger adults [81], [82], and the only study in adults over sixty, including amnestic mild cognitive impairment, characterized cerebral oxygenation and tolerance rather than cognition [83]. To my knowledge, and within the controlled acute-hypoxia literature reviewed here, no study has yet measured cognitive performance in healthy older adults or compared younger and older adults within a single protocol, so the associative and binding-dependent deficits of central interest here remain unexamined under controlled hypoxia in the group to which the inference is meant to apply. Second, associative or relational memory is rarely the explicit target; where it has been probed, acute hypoxia has produced either no change or altered interference rather than a simple decrement [27], [84]. Third, end-tidal or arterial CO₂ is rarely measured or controlled, even though it strongly modulates the cognitive effect [24], [79].

**Table 1:** Representative acute hypoxia protocols from the cognitive literature.

Study	Hypoxia exposure (type, FiO₂, duration, onset/reoxygenation)	Population	Targeted cognitive domain	Key outcomes	Capnic status
Gatti et al. [27]	Normobaric hypoxic tent (Study 1), FiO₂ ≈ 20.9 / 15.1 / 13.6%; real high altitude, hypobaric (Study 2); brief laboratory and multi-day field exposures; field onset and reoxygenation gradual	Young adults (Study 1, *n* = 18; Study 2, *n* = 20)	Associative memory for emotional stimuli; cerebral oxygenation (fNIRS)	No significant memory difference in normobaric Study 1; in the field, an emotional-memory advantage at low altitude reversed at high altitude, with concurrent changes in cerebral oxygenation	Not reported
Loprinzi et al. [84]	Normobaric; FiO₂ 0.12; 30 min; abrupt onset and reoxygenation	Young adults (*n* = 21; ≈ 21 y)	Associative memory (AB/AC paired-associate interference)	Acute hypoxia reduced retroactive interference (condition × time interaction), a possible facilitation of associative memory rather than a decrement	Not reported
Williams et al. [58]	Normobaric, graded; FiO₂ 0.209 / 0.17 / 0.145 / 0.12; 60 min per condition; abrupt onset and reoxygenation	Young healthy males (*n* = 12; ≈ 22 y)	Working memory (n-back); attention	n-back accuracy fell at FiO₂ 0.12 (≈ 82% vs 93%); the change tracked falls in SpO₂ and cerebral oxygenation, but not catecholamines or cortisol	Not reported or controlled
Friend et al. [79]	Normobaric; isocapnic vs poikilocapnic hypoxia (PETO₂ ≈ 45 mmHg) and euoxic hypocapnia; ≈ 1 h per condition; abrupt onset	Young healthy adults (n = 20; male subgroup)	Reaction time; spatial working memory	Isocapnic hypoxia did not slow reaction time, whereas poikilocapnic hypoxia and hypocapnia did, indicating that hypocapnia rather than hypoxaemia per se drove slowing; working memory unaffected	Explicitly measured and clamped (end-tidal control)
Turner et al. [29]	Normobaric; FiO₂ ≈ 10% vs 21% sham; 50 min; abrupt onset and reoxygenation; single-blind, matched-pairs	Young healthy adults (*n* = 22)	Broad battery (memory, attention, executive function)	SpO₂ fell by ≈ 20%; broad and substantial impairment (e.g., composite and verbal memory reduced by roughly a third) and normal practice gains blocked	Not reported
Davranche et al. [57]	Hypobaric field (rapid helicopter ascent to 4350 m); 4 days; abrupt onset; gradual descent	Adult males (*n* = 11)	Executive control (conflict task); time perception; prefrontal fNIRS	On arrival, reaction time slower and decision errors roughly doubled; the slowing resolved by day 2 whereas the error rate remained elevated; prefrontal fNIRS responses increased from day 0 to day 2, consistent with compensation; internal-clock slowing observed.	Not reported
de Aquino Lemos et al. [47]	Normobaric chamber; simulated 4500 m; 24 h	Young adult males (*n* = 10; 23–30 y)	Attention, working and visual memory, executive function, processing speed; mood	Sleep reduced and depressive mood, anger, and fatigue increased, alongside broad cognitive slowing, illustrating non-specific state changes that accompany hypoxia	Not reported
Nation et al. [28]	Hypobaric chamber; simulated 20,000 ft.; brief (≈ 15 min at altitude); abrupt ascent	Military aircrew (*n* = 9–17 per experiment; mean age 31 y, 22–48; mostly male)	Learning and memory; attention	Rapid impairment of learning and memory with attention relatively preserved; encoding and retention affected, indicating selective memory vulnerability under brief severe hypoxia	Neither SpO₂ nor CO₂ measured
Boulares et al. [81]	Normobaric, graded; FiO₂ 20.9 / 17.4 / 14.5 / 12.5%; 45–65 min per session; abrupt onset and reoxygenation	Middle-aged healthy adults (*n* = 16)	Executive function (Stroop, Operation Span); working memory (n-back); visuospatial span (Corsi); prefrontal fNIRS	From FiO₂ 14.5%, reaction time slowed and cognitive-flexibility accuracy and complex working memory declined; 2-back declined only at 12.5%; visuospatial span unaffected; prefrontal oxygenation fell progressively	Not reported or controlled
Cortez et al. [83]	Normobaric; FiO₂ 0.10; brief (≈ 4.5 min) with room-air recovery; abrupt onset and reoxygenation	Older adults (71 ± 6 y), incl. Amnestic MCI (*n* = 32 aMCI, 35 controls)	Cerebral oxygenation and tolerance only (no cognitive task)	Arterial saturation fell to ≈ 77%; prefrontal oxygenation lower at baseline in aMCI, but hypoxia and recovery dynamics were similar to controls; brief moderate hypoxia well tolerated	CO₂ measured (not clamped)

*Note.* FiO₂ = fraction of inspired oxygen; SpO₂ = peripheral oxygen saturation; PETO₂/PETCO₂ = end-tidal oxygen or carbon dioxide; fNIRS = functional near-infrared spectroscopy; aMCI = amnestic mild cognitive impairment. The selection is illustrative rather than exhaustive.

### 5.2 Phenotype specificity and dissociation logic

A meaningful oxygen-limitation probe should not merely produce a global decrement; it should reproduce a dissociation that is diagnostically relevant for cognitive aging. A prime candidate is the associative deficit, in which older adults show disproportionate impairment for associative or binding-dependent memory relative to item memory [34], [35], [85]. This dissociation should be evaluated as an explicit condition-by-memory-type interaction instead of being inferred from separate main effects. Because associative outcomes can reflect changes in sensitivity, bias, or strategy, interpretations should also remain at the level of decision processes where possible. If emotional material is included, expectations must remain anchored to the arousal-and-binding literature. Emotional arousal can sharpen item processing while leaving associative binding unchanged or impaired, so emotional effects are informative only when they remain distinguishable from arousal-mediated interference [42], [43], [44], [45].

### 5.3 Coupling to oxygen-sensitive markers

The core inferential claim is that hypoxia-induced cognitive change reflects oxygen limitation rather than merely occurring in a hypoxic context. Observed cognitive dissociations should therefore covary, at least partially, with oxygen-sensitive physiological indices after plausible alternatives such as CO₂-related hemodynamic shifts have been considered. SpO₂ provides a minimal marker of achieved challenge, and cerebral oxygenation proxies can strengthen inference when available, but the theoretical requirement is coupling to an oxygen-relevant signal rather than commitment to any single measurement modality. Because peripheral and cerebral indices are imperfect and context-sensitive, mechanistic support should be interpreted convergently rather than absolutistically.

### 5.4 Discriminant validity against nonspecific state change

Acute hypoxia can alter discomfort, anxiety, fatigue, sleepiness, and perceived effort (e.g., [47], [48]), so an apparent cognitive effect is not automatically an oxygen effect. Theoretical support is strongest when the predicted cognitive dissociation is more closely tied to oxygen-relevant indices than to expectancy, generic stress, or broad arousal-related alternatives, and when competing physiological drivers such as CO₂-related changes are explicitly considered. The same logic applies to emotional material. A phenotype counts as aging-relevant here only when it shows the predicted dissociation, couples to oxygen-sensitive variation, and cannot be explained equally well by measured non-specific changes in stress, discomfort, or fatigue.

### 5.5 An illustrative laboratory implementation

These conditions can be translated into a concrete, if deliberately simplified, laboratory study. A within-participant design would expose healthy adults to at least two levels of dose-controlled normobaric hypoxia and a normoxic sham, with the achieved challenge verified continuously through SpO₂ and, where possible, cerebral oxygenation, and with end-tidal CO₂ monitored so that oxygen-content effects can be separated from hypocapnia-related hemodynamic shifts [58], [79]. The cognitive endpoint would be an associative memory task that yields item and relational measures from the same materials, so that the diagnostic dissociation can be tested as an explicit condition-by-memory-type interaction rather than inferred from separate scores [34], [35]. Encoding under hypoxia with delayed retrieval after reoxygenation would allow the predicted recovery to be examined against an endpoint-specific window defined in advance [21]. Because the timing of the hypoxic window relative to the task isolates different processes, encoding under hypoxia, retrieval under hypoxia, and retrieval after reoxygenation should be distinguished: the first asks whether oxygen limitation impairs the formation of new associations, the second whether it impairs their retrieval, and the third whether any deficit recovers once oxygenation is restored. To establish discriminant validity, the protocol would also record discomfort, anxiety, fatigue, sleepiness, and perceived effort, so that an apparent associative cost can be evaluated against non-specific state change rather than assumed to reflect oxygen limitation [47], [48]. Because the inference concerns cognitive aging, the paradigm connects to aging in two complementary ways. The most direct is convergence. If oxygen limitation contributes to the associative deficits of later life, young adults under hypoxia should reproduce the qualitative profile of older adults at rest, with a disproportionate relational binding deficit and relative sparing of item memory [34], [35]. This comparison is informative before any older adult is exposed to hypoxia, since it sets young-adult hypoxia against the well-characterized resting profile of aging, and it sidesteps the dosing and safety problems that hypoxia in older adults would raise. The complementary but less direct route is differential vulnerability. If cerebrovascular reserve narrows with age, a matched dose should impair older adults more than younger adults, provided the dose is equated at the level of the delivered insult, for example matched SpO₂ or cerebral oxygenation, and not inspired fraction. An age-related reduction in oxygen extraction reserve has not been established [6], and no published study has compared, at matched arterial desaturation, the cognitive effect of acute normobaric hypoxia in younger and older adults, so this vulnerability prediction remains untested. Its physiological plausibility is supported by greater cerebral vulnerability to hypoxia with age [86] and by executive and working-memory costs that emerge at a milder hypoxic dose in middle-aged than in younger adults [81], [82]. A first study in young adults, in whom controlled hypoxia is best characterized, would establish the paradigm, the predicted dissociation, and the convergence with the resting aging profile; extending it to older adults, who require additional screening, safety monitoring, and ethical oversight, would then test the vulnerability prediction directly (see Section 6.4).

## 6 Boundaries and limitations

Several boundaries follow directly from the logic of the proposal and should constrain interpretation from the outset. Here, boundaries denote the conditions that determine when the oxygen-limitation inference applies, whereas limitations denote constraints of the present approach and the available evidence.

### 6.1 Acute hypoxia is a probe, not a model of aging

Acute normobaric hypoxia is not a model of aging. Aging involves chronic structural and network-level changes, including gray- and white-matter alterations, synaptic change, and neurotransmitter shifts, that accumulate over decades and cannot be recapitulated by a brief oxygen manipulation [87], [88]. Even if hypoxia in young adults produces an aging-like dissociation, that result would support a shared constraint pathway rather than mechanistic equivalence between hypoxia and aging. An important asymmetry should also be acknowledged. Acute normobaric hypoxia lowers arterial oxygen content and often elicits compensatory vasodilation and higher CBF, whereas later-life vascular aging more often involves reduced perfusion, prolonged transit, and compensatory OEF elevation. The proposed bridge is therefore the narrower claim that both states can reduce cerebrovascular reserve and thereby constrain the capacity to meet transient metabolic demand during high-load cognition, not hemodynamic identity.

### 6.2 CO₂ physiology, compensation, and barometric context

Oxygen restriction is not physiologically pure. Hypoxemia triggers compensatory cerebrovascular responses that can partially preserve oxygen delivery, and hypoxia commonly induces hyperventilation and reductions in PETCO₂; hypocapnia can in turn reduce cerebral blood flow and mimic or amplify apparent oxygen effects if it is not measured and modeled [19], [77], [78], [79]. Findings obtained under normobaric laboratory hypoxia also do not automatically generalize to hypobaric chamber or field altitude exposure, which can differ in barometric context and symptom profile [75], [76]. More broadly, heterogeneity in dose, duration, and acclimatization is a major reason why cognitive effects vary in magnitude and selectivity across the literature [23], [89]. Task type and sensitivity are a further reason for this variability, so null results can reflect task insensitivity, not the absence of an effect [23], [24], [89].

### 6.3 Recovery kinetics, measurement constraints, and inferential restraint

Recovery kinetics and measurement constraints require similar restraint. Although brief hypoxic exposures often show rapid recovery after reoxygenation, some cognitive and operational impairments can persist beyond immediate physiological normalization in certain contexts, depending on dose, duration, and endpoint [21]. Cerebral oxygenation proxies are also vulnerable to systemic physiology and extracerebral contamination, and the same caution extends to autonomic and electrophysiological indices, whose relation to hypoxia varies with severity, respiratory patterning, and task context [63], [74]. Finally, because the proposed logic relies on dissociations and coupling to physiology, null or weak findings may reflect assay insensitivity, ceiling or floor effects, or shifts in response strategy rather than the absence of an oxygen-related mechanism.

### 6.4 Population and generalizability limits

Population and generalizability limits should also be made explicit. The present framework concerns brief, laboratory-controlled hypoxia in healthy young and healthy older adults. Generalization to populations with cerebrovascular disease, respiratory compromise, or neurodegenerative conditions is limited and requires adapted safety procedures and medical oversight. Chronic intermittent hypoxemia in conditions such as obstructive sleep apnea is mechanistically distinct from brief laboratory hypoxia, even if hypoxic burden is associated with hippocampal atrophy and cognitive impairment in affected populations [90], [91]. External validity is additionally constrained by the predominance of WEIRD (Western, Educated, Industrialized, Rich, and Democratic) samples in both cognitive aging and hypoxia research [92]. Sex and hormonal status may also moderate hypoxia sensitivity and cognitive aging trajectories through respiratory, autonomic, and neurovascular pathways, and should therefore be treated as potential moderators instead of background noise [93], [94], [95].

## 7 Broader implications

### 7.1 A causal bridge between vascular physiology and selective cognitive aging

If these conditions are met, acute normobaric hypoxia offers a way to move from descriptive aging differences toward more specific causal tests of whether oxygen-delivery and cerebrovascular-reserve constraints contribute to selected cognitive phenotypes. The strongest target remains binding-dependent episodic memory, where age effects are reliably larger for associative than for item memory [34], [35]. In that sense, the scientific value of the framework is not limited to positive findings. Supportive results would strengthen oxygen-limitation accounts, whereas global impairment or state-driven explanations would narrow their scope and sharpen mechanistic theory.

### 7.2 Sharper endpoints for intervention and stratification

The framework also suggests sharper cognitive endpoints for intervention research. If hypoxia-induced associative deficits are shown to covary with oxygen-sensitive or capnia-sensitive indices, interventions that improve cerebrovascular reserve or oxygen delivery should be expected to attenuate the same dissociations. This logic could help refine mechanistic work on exercise and other vascular-health interventions, where improvements in memory are often reported but the operative pathways remain incompletely specified [96], [97]. Inter-individual variability in desaturation, ventilatory response, and cerebral oxygenation compensation may also become theoretically informative rather than being treated only as nuisance variance, helping to identify individuals with lower cerebrovascular reserve [61], [98].

### 7.3 Delimiting the explanatory scope of hypoxia in cognitive aging

More broadly, the framework helps delimit what hypoxia can and cannot explain in cognitive aging. A common interpretive risk in the broader literature is to over-attribute broad hypoxia-related performance decrements to aging-like mechanisms. By requiring phenotype specificity, coupling to oxygen-sensitive variation, and discriminant validity against non-specific state change, the present argument is intended to prevent premature conclusions. A negative outcome would still be theoretically valuable if it ruled out the predicted dissociation under well-characterized hypoxia, thereby redirecting attention toward alternative aging mechanisms [87], [88].

## 8 Conclusion

Acute normobaric hypoxia may serve as a reversible probe of oxygen-delivery and cerebrovascular-reserve constraints relevant to selected aspects of cognitive aging, especially when inference is anchored to associative-memory dissociations and explicit physiological characterization. Its value does not depend on treating hypoxia as a model of aging or as a substitute for calibrated metabolic imaging. Rather, its value lies in offering a tractable causal probe whose interpretability depends on dose and capnic characterization, phenotype specificity, coupling to oxygen-sensitive markers, and discriminant validity against non-specific state change.

## CRediT authorship contribution statement

**Pierrick Laulan:** Writing – review & editing, Writing – original draft, Validation, Resources, Investigation, Funding acquisition, Conceptualization.

## Consent for publication

Not applicable.

## Declaration of generative AI and AI-assisted technologies in the writing process

During the preparation of this work, generative AI was used to assist with language editing and clarity. The author reviewed and edited the content as needed and takes full responsibility for the content of the publication.

## Funding

This work was supported by the 10.13039/501100001711Swiss National Science Foundation (SNSF), grant CRSK-1_237841.

## Declaration of competing interest

The author declares that he have no known competing financial interests or personal relationships that could have appeared to influence the work reported in this paper.
